# Linkage Between miR‐218‐2 (rs11134527) Genetic Polymorphism and Breast Cancer Risk: A Case‐Control Study in the Bangladeshi Women

**DOI:** 10.1002/hsr2.72092

**Published:** 2026-04-16

**Authors:** A. M. Shamim, Aiman Hossan, Md. Shafiul Hossen, Md Abdul Barek, Mohammad A. Rashid, Mohammad Safiqul Islam

**Affiliations:** ^1^ State University of Bangladesh South Purbachal, Kanchan Dhaka Bangladesh; ^2^ Department of Pharmacy, Southern University Bangladesh Arefin Nagar, Baizid Bostami Chattogram Bangladesh; ^3^ Department of Pharmacy State University of Bangladesh, South Purbachal, Kanchan Dhaka Bangladesh; ^4^ Department of Pharmacy Noakhali Science and Technology University Noakhali Bangladesh; ^5^ Department of Pharmaceutical Chemistry Faculty of Pharmacy, University of Dhaka Dhaka Bangladesh

**Keywords:** Bangladeshi population, breast cancer, genetic susceptibility, miR‐218‐2, rs11134527 polymorphism, T‐ARMS‐PCR

## Abstract

**Background and Aims:**

The growth and spread of breast carcinoma are influenced by genetic factors. Diverse forms of cancer have been reported to display multiple subtypes of the *microRNA* gene. Therefore, the current study aimed to explore the connection between the *miR‐218‐2* (rs11134527) and breast cancer risk.

**Methods:**

A total of 303 participants (158 breast cancer patients and 145 healthy controls) were enrolled. Clinical and demographic data were collected through structured questionnaires and hospital records. Genotyping of miR‐218‐2 rs11134527 was performed using the T‐ARMS‐PCR technique. Statistical analyses were conducted with SPSS v25.0 and MedCalc v19.0.7. The adjusted odds ratio (aOR) using binary logistic regression that controls for age and BMI was employed to investigate the relationship between the targeted SNPs and the risk of breast cancer.

**Results:**

Among the patients, invasive ductal carcinoma was the most frequent histological type (49.61%), followed by lobular carcinoma (17.22%). Grade II tumors (63.75%) were predominant. Ultrasound (64.18%) and biopsy (68.66%) were the most common diagnostic tools. Chemotherapy was the principal treatment (62.12%), with cyclophosphamide (69.62%), doxorubicin (53.80%), and paclitaxel (56.33%) as the most prescribed agents. Genotype analysis revealed that individuals carrying the AA genotype had a significantly higher risk of breast cancer than those with GG (additive model 2: OR = 2.48, 95% CI = 1.12–5.48, *p* = 0.025). Significant associations were also observed under the recessive model (AA vs. GG + AG: OR = 1.96, 95% CI = 1.0–3.86, *p* = 0.051) and the allelic model (A vs G: OR = 1.59, 95% CI = 1.06–2.39, *p* = 0.026).

**Conclusion:**

The *miR‐218‐2* rs11134527 A allele confers an increased risk of breast cancer in Bangladeshi women, supporting its potential role as a population‐specific genetic biomarker for susceptibility assessment.

**Abbreviations**
3'UTR: 3'‐untranslated region
BC: Breast Cancer
CI: Confidence Interval
CT: Computed Tomography
EDTA: Ethylenediaminetetraacetic acid
ER: Estrogen Receptor
FNAC: Fine Needle Aspiration Cytology
GWAS: Genome‐Wide Association Studies
HER2: Human Epidermal Growth Factor Receptor 2
HWE: Hardy–Weinberg Equilibrium
MgCl2: Magnesium Dichloride
miR: MicroRNA
OR: Odds Ratio
PR: Progesterone Receptor
SNPs: Single Nucleotide Polymorphisms
T‐ARMS‐PCR: Tetra‐Primer Amplification Refractory Mutation System‐Polymerase Chain Reaction
USGs: Ultrasounds

## 1 Introduction

Recently, breast cancer (BC) has been identified as one of the most prevalent carcinomas in females. In 2020, the estimated number of BC patients was over 2 million, representing roughly 11.7% of all cancer cases globally. Furthermore, it is believed to be the fifth most frequent contributor to cancer‐related deaths globally (684,996 deaths, 6.9%) [1]. The growing rate of BC occurrence is also considered an alarming concern in Bangladesh, where more than 22 of every 100,000 women are affected by breast carcinoma [2]. Approximately 12,764 new patients of breast cancer are registered in Bangladesh each year, with 6846 of those cases ending in death [3]. The world health crisis is now being caused by the startling increase in cancer incidence. This deadly illness affects women from different countries of low to high income as well as women of different ethnicities because it knows no geographical bounds [4].

The interplay of genetic makeup and environmental exposures plays a key role in the development of breast cancer [5]. The predisposition to cancer is caused by a sensitive gene that experiences genetic variation. It has been determined that 5%–10% of all breast cancer patients have a genetic variation in a sensitive gene. Therefore, in recent decades, gene mutation has been considered the most important risk factor for breast cancer [6]. Nevertheless, the molecular mechanisms underlying carcinogenesis remain unknown. The incidence frequency of BC can be reduced by determining the genetic factors that may instigate the onset of the disease [7]. The genome‐wide association studies (GWAS) have discovered a large number of single‐nucleotide polymorphisms (SNPs) of different genes that are connected to the development of BC [8]. Similarly, previous studies have shown that microRNAs play a role in the onset of cancer [9].

MicroRNAs, also known as post‐transcriptional gene regulators, play a crucial role in both normal and abnormal cellular processes [1]. These serve as the fundamental building blocks of the core tenets of molecular biology. Additionally, they are widely distributed throughout the high eukaryotic cells' genomes [10, 11]. Furthermore, it has been demonstrated that this gene regulates the steps of gene activation and suppression. In multiple signaling pathways, they are also capable of coordinating. Conversely, several miRNA subtypes can work together to control a single miRNA target. This diverse function contributes to the intricacy of gene regulation, which in turn mediates the intricate network of eukaryotic cell function [12] encompassing the differentiation of hematopoietic cells, apoptosis, cell division, and organ development [13].

Moreover, gene expression of multiple *microRNA* gene subtypes has been detected in various cancer types. It has been discovered that overexpression of several subtypes, including miR‐21, miR‐210, and miR‐221, occurs in triple‐negative breast cancer [14, 15]. The MIR218‐2 gene (microRNA 218‐2), located on 5q35.1 within the SLIT3 gene, encodes hsa‐miR‐218‐2, a microRNA implicated in tumor suppression. The polymorphism of *miR‐218‐2* (rs11134527) and its association with carcinoma risk have been the subject of numerous studies in different ethnic groups [5]; however, no case‐control study has been conducted in the Bangladeshi population. In order to thoroughly investigate any potential genetic correlations between Bangladeshi women's risk of breast cancer and the *miR‐218‐2* rs11134527 polymorphism, we designed a study. Based on genetic variation, we can speculate that the BC in the Bangladeshi population may be linked to the *miR‐218‐2* rs11134527 polymorphism.

## 2 Methods and Materials

### 2.1 Population Study

Participants in this case‐control study included 303 Bangladeshi women, comprising 158 cases and 145 controls. Individuals in good health served as controls, and BC patients were considered cases. The study received ethical approval from the Ethics Committee of the National Institute of Cancer Research and Hospital (approval no. NCRH/Ethics/2019/446). Experimental work was carried out at the Pharmacogenomics Research Laboratory, Noakhali Science and Technology University, Noakhali, Bangladesh. Before taking their blood samples, the participants were given a written consent form outlining the goals of the study. Additionally, individuals with long‐term comorbidities such as liver disease and kidney disorders were not allowed to participate in our research. We also excluded the healthy individuals with a family history of cancer from our study. Additionally, our volunteers collected all data related to the clinicopathological characteristics, patient demographics, diagnosis, and medication history of breast cancer patients.

### 2.2 DNA Extraction and Primer Design

Around 3 ml of blood sample was withdrawn from each participant. The extracted blood was then put into a plastic sterile tube containing EDTA‐Na2. The sample tube was kept in the refrigerator at −80° C until the DNA was extracted. Genomic DNA was collected using an approved procedure that is consistently performed in our laboratory [16]. The purity of the isolated DNA was evaluated using a micro volume spectrophotometer (Genova Nano, Jenway) set at a ratio of 260 nm to 280 nm. The intended primer sequence was designed using the Primer1 online programme. To amplify the desired alleles, we employed outer (forward and reverse) and inner (forward and reverse) primers. Table S1 shows the primer sequences that we used in our research.

### 2.3 Genotyping

The polymerase chain reaction‐based tetra primer amplification refractory mutation system (T‐PCR‐ARMS) method was used to genotype the targeted polymorphism of the *miRNA‐218‐2* gene. Equipped with MgCl2, nuclease‐free water, and an adequate quantity of primers, the emeraldAmp GT PCR master mix was used to create the PCR premix. Primers for outer and inner forward and reverse (1.5 µl and 2.5 µl, respectively) and the PCR premix solution were combined to create approximately 120 µl of PCR master mix solution. For twelve samples (10 µl/reaction), the PCR master mix solution was prepared. One microliter of the DNA sample was added to 10 milliliters of the premix to initiate the PCR process. Primer melting temperatures were calculated using Primer1 software, and PCR amplification was performed at an optimized annealing temperature of 57°C. All reactions were conducted using EmeraldAmp GT PCR Master Mix (Takara), which supplies a final MgCl₂ concentration of 2.5 mM. Approximately 1% agarose gel electrophoresis, stained with ethidium bromide, was used to confirm the PCR product after the reaction was terminated. DNA bands specific to each allele were identified using the predicted fragment lengths and properties of the targeted SNPs. The PCR conditions and fragment lengths for the targeted SNPs are shown in Tables S1 and S2.

### 2.4 Statistical Analysis

Utilizing the Chi‐square (χ2) test, the Hardy–Weinberg Equilibrium (HWE) was verified. The odds ratio (OR) with a 95% confidence interval (CI) was estimated to explore the association with risk. Allelic and genetic frequencies were expressed as percentages. The adjusted odds ratio (aOR) using binary logistic regression that controls for age and BMI was employed to investigate the relationship between the targeted SNPs and the risk of breast cancer. The threshold of 0.05 was set for a significant correlation.

## 3 Results

### 3.1 Demographic Distribution of Participants

The demographic and clinicopathological characteristics of the patients and controls who took part in our study are displayed in Table 1. Age, BMI, and marital status were our demographic variables. Patients with breast cancer were 46.71 years old on average. Around sixty‐two percent of the patients in our study were over 45 years old. The percentage of patients under 45 was only 36.71%. Additionally, we have gathered approximately 48.97% of healthy participants who are 45 years of age or older. The control group's mean age was 36.17 years. The patients' average body mass index (BMI) was 25.19, whereas that of the healthy volunteers was 22.01. In our study, married individuals made up the majority of both patients and healthy volunteers (88.61% and 87.59%, respectively).

**Table 1:** Distribution of demographic and clinicopathological variables of participants.

Variables	Cases (*n* = 158) *N* (%)	Controls (*n* = 145) *N* (%)
**Age (years)**		
< 45	58 (36.71)	74 (51.03)
45–60	80 (50.3)	57 (39.31)
> 60	20 (12.66)	14 (9.66)
45–60 + > 60	100 (63.29)	71 (48.97)
**Mean Age (years)**		
Minimum Age	27	25
Maximum Age	71	65
Average ± SD	46.71 ± 9.92	36.17 ± 11.87
**BMI (kg/m** ^**2**^ **)**		
Average ± SD	25.29 ± 2.62	22.01 ± 1.69
**Marital Status**		
Married	140 (88.61)	127 (87.59)
Unmarried	18 (11.39)	18 (12.41)
**Type of BC**		
Atypical ductal hyperplasia	9 (7.09)	N/A
Duct cell carcinoma	9 (7.09)	N/A
Infiltrating duct cell carcinoma	26 (20.47)	N/A
Intraductal carcinoma	4 (3.15)	N/A
Invasive duct cell carcinoma	63 (49.61)	N/A
Medullary carcinoma	4 (3.15)	N/A
Metastatic duct cell carcinoma	6 (4.72)	N/A
Triple negative BC	6 (4.72)	N/A
**Grade of BC**		
Ⅰ	13 (16.25)	N/A
Ⅱ	51 (63.75)	N/A
Ⅲ	16 (20.00)	N/A
**ER, PR, HER2 Status**		
ER (+)	50 (42.02)	N/A
ER (−)	59(49.58)	N/A
PR (+)	64 (53.78)	N/A
PR (−)	55 (46.23)	N/A
HER2 (+)	43 (36.13)	N/A
HER2 (−)	66(55.46)	N/A
Triple negative	7 (5.88)	N/A

*Note:* N/A stands for Not Applicable; *p* < 0.05 was considered a statistically significant.

The types, grades, ER, PR, and HER2 status of breast cancer were assessed in the case of clinicopathological parameters. Invasive duct cell carcinoma was the most common type of breast cancer in patients (49.61%), despite the fact that patients recruited for our study had a variety of breast cancer types as listed in Table 1. Among patients with a diagnosis of breast cancer, infiltrating duct cell carcinoma was also frequently found (20.47%). Patients also had triple‐negative BC, metastatic ductal carcinoma, and atypical ductal hyperplasia. Most of the patients with data on the grade of breast cancer were identified with grade II breast cancer (63.75%). Around 119 of 158 patients were recognized as having ER, PR, and HER2 status. Among them, ER, PR, and HER2‐positive status were found in 42.02%, 53.78%, and 36.13%, respectively, of patients. Around 49.58%, 46.23%, and 55.46% of patients had ER, PR, and HER2‐negative status, respectively.

### 3.2 Diagnosis and Current Treatment Scenario of Breast Cancer Patients in Bangladesh

Table 2 provides an overview of the current treatment regimen and diagnosis process for patients diagnosed with breast cancer in Bangladesh. The most common methods of diagnosis for locating breast cancer were biopsies and ultrasounds (USGs). A biopsy revealed breast cancer in roughly 68.66% of patients, while a USG revealed the disease in 64.18%. In order to detect breast cancer, other standard diagnostic techniques include computed tomography (CT), fine needle aspiration cytology (FNAC), and X‐ray. These methods were used by 37.31%, 31.34%, and 25.37% of patients, respectively, to screen for cancer. Chemotherapy was the most often used treatment for breast cancer in terms of treatment patterns. In approximately 62.12% of cases, chemotherapy was used as a treatment. About 54.88% of patients receiving chemotherapy were found to be on cycles 4 through 8. Of the patients, 39.02% were identified as having chemotherapy cycles 1–3. With percentages of 25.00%, 15.15%, and 12.88%, surgery, mastectomy, and radiation therapy were also common treatment patterns for patients with breast cancer. Table 2 also provides the names of several medications used to treat breast cancer. The most often prescribed medications for the treatment of breast cancer in Bangladesh were doxorubicin (53.80%), paclitaxel (56.33%), and cyclophosphamide (69.62%). Additionally, percentages for dexamethasone, cisplatin, epirubicin, filgrastim, and 5‐FU were discovered to be 27.85%, 17.09%, 12.03%, and 17.09%, respectively.

**Table 2:** Diagnosis and current treatment history of BC patients.

Variables	Cases (*n* = 158) *N* (%)	Controls (*n* = 145) *N* (%)
**Diagnosis**		
Biopsy	92(68.66)	N/A
Cancer Antigen 15‐3 (CA 15‐3)	5 (3.73)	N/A
Computed Tomograpghy (CT)	34 (25.37)	N/A
X‐Ray	50 (37.31)	N/A
Echocardiogram	29(21.64)	N/A
Fine Needle Aspiration Cytology (FNAC)	42 (31.34)	N/A
Ultrasound (USG)	86 (64.18)	N/A
**Treatment**		
Chemotherapy	82 (62.12)	N/A
Mastectomy	20 (15.15)	N/A
Lumpectomy	6 (4.55)	
MRM	10 (7.58)	N/A
Radiation therapy	17 (12.88)	N/A
Surgery	33 (25.00)	N/A
**Chemotherapy Cycle**		
1–3	32 (39.02)	N/A
4–8	45(54.88)	N/A
9–12	5 (6.10)	N/A
**Drug Name**		
5‐FU	19 (12.03)	N/A
Carboplatin	12 (7.60)	N/A
Cisplatin	27 (17.09)	N/A
Cyclophosphamide	110 (69.62)	N/A
Dexamethasone	44 (27.85)	N/A
Docetaxel	5 (3.16)	N/A
Doxorubicin	85 (53.80)	N/A
Epirubicin	19 (12.03)	N/A
Filgrastim	27 (17.09)	N/A
Gemcitabine	5 (3.16)	N/A
Herceptin	12 (7.60)	N/A
Paclitaxel	89 (56.33)	N/A
Tamoxifen	12 (7.60)	N/A

*Note:* N/A stands for Not Applicable.

### 3.3 Genotypic Association of miR‐218‐2 rs11134527 Variants With Breast Cancer

Table 3 shows the genotypic connection between the *miR‐218‐2 rs11134527* variants and the risk of BC in Bangladeshi females. The present study found that about 23.42%, 41.77%, and 34.81% of breast cancer patients were homozygotes (GG), heterozygotes (AG), and mutant homozygotes (AA), respectively. In contrast, the frequencies in the group of healthy volunteers were approximately 35.17%, 42.76%, and 22.07%. Hardy–Weinberg Equilibrium (HWE) was also not violated by the genotypic distribution (*p* > 0.05) of both cases (χ^2^ = 3.726) and controls (χ^2^ = 2.446). We also investigated the genetic associations between the miR‐218‐2 rs11134527 variant and breast cancer risk by employing a range of genetic inheritance models, including additive, dominant, over‐dominant, recessive, and allelic models. Regarding the additive model, the homozygotes that were mutant and heterozygotes were compared to the homozygotes that were normal. This led to the classification of the additive model into two types: additive model 1 (AG vs. GG) and additive model 2 (AA vs. GG). The rs11134527 polymorphism was found to have a strong correlation with an increased BC risk in the additive model 2 (OR = 2.48, CI = 1.12–5.48, *p* = 0.025). Nonetheless, no significant correlation was found between the polymorphism and the BC risk in additive model 1 (OR = 1.61, CI = 0.73–3.52, *p* = 0.237). In the current study, the strong connection between the rs11134527 genetic variant and the susceptibility to breast cancer in the recessive (AA vs. GG + AG: OR = 1.96, CI = 1.0–3.86, *p* = 0.051), and allelic (A vs. G: OR = 1.59, CI = 1.06–2.39, *p* = 0.026) models was also investigated. However, no strong evidence was found between the rs11134527 polymorphisms and elevated BC risk in both the dominant (AG + AA vs. GG: OR = 1.77, CI = 1.07–2.93, *p* = 0.025) and the over‐dominant (AG vs. GG + AA: OR = 0.98, CI = 0.52–1.82, *p* = 0.938) model.

**Table 3:** Genotypic association of miRNA‐218‐2 rsVariants with breast cancer.

Genetic models	Genotype/Allele	Cases (*n* = 158)	HWE		Controls (*n* = 145)	HWE		Risk Analysis	
			χ2	*p* value		χ2	*p* value	aOR(95% Cl)	*p* value
	GG	37 (23.42%)	3.726	0.054	51 (35.17%)	2.446	0.118	1	
Additive model 1 (AG vs. GG)	AG	66 (41.77%)			62 (42.76%)			1.61 (0.73–3.52)	0.237
Additive model 2 (AA vs. GG)	AA	55 (34.81%)			32 (22.07%)			2.48 (1.12–5.48)	**0.025**
Dominant model (AG + AA vs. GG)	GG	37 (23.42%)			51 (35.17%)			1	
	AG + AA	121 (76.58%)			94 (64.83%)			1.89 (0.95–3.75)	0.069
Recessive model (AA vs. GG + AG)	GG + AG	103 (65.19%)			113 (77.93%)			1	
	AA	55 (34.81%)			32 (22.07%)			1.96 (1.0–3.86)	**0.051**
Over‐dominant model (AG vs. GG + AA)	GG + AA	92 (58.23%)			83 (57.24%)			1	
	AG	66 (41.77%)			62 (42.76%)			0.98 (0.52–1.82)	0.938
Allele (A vs. G)	G	140 (44.30%)			164 (56.55%)			1	
	A	176 (55.70%)			126 (43.45%)			1.59 (1.06–2.39)	**0.026**

*Note: p* < 0.05 was considered statistically significant.

Abbreviation: OR = Odds Ratio.

### 3.4 In‐Silico Gene Expression Analysis of MIR218‐2

**Figure 1:**
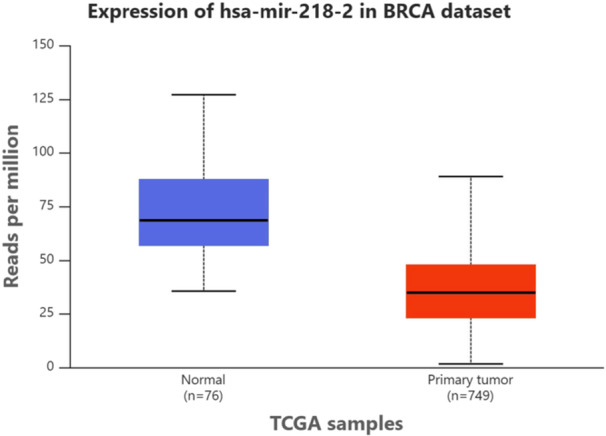
Expression levels of hsa‐miR‐218‐2 in Normal versus Tumor tissues (*p* = 1.62 × 10^−12^) (Data retrieved from the UALCAN database).

To complement our genotyping findings, we investigated the expression levels of MIR218‐2 (hsa‐miR‐218‐2) in breast cancer and corresponding normal tissues through analysis of the publicly available UALCAN database, which incorporates TCGA data. The analysis revealed that hsa‐miR‐218‐2 expression was markedly reduced in breast cancer samples compared with controls (*p* = 1.62 × 10⁻¹²) (Figure 1). This marked reduction supports the tumor‐suppressive role of miR‐218 reported in earlier studies and aligns with our genetic association results, suggesting that the rs11134527 polymorphism in MIR218‐2 may influence susceptibility to breast cancer through regulatory effects on miRNA expression.

## 4 Discussion

The death rate from cancer is currently rising dangerously on a global scale. Cancer is also becoming increasingly prevalent over time [17]. According to research projections, this trend is expected to result in approximately 30 million cancer patients by 2040 [18]. In the world, breast cancer is thought to be the primary cause of death for women [19]. Furthermore, genetic variations in different genes also play a role in the development of this terrible illness. Therefore, knowledge of genetic alterations in the genes associated with the appearance of carcinoma of the breast is crucial in order to find out their function in the progression of cancer. The genetic association analysis may help those who have breast cancer to choose appropriate healthcare and treatment decisions [20]. The current study set out to ascertain whether Bangladesh's genetic variant miR‐218‐2 (rs11134527) and its susceptibility to breast cancer are related.

MicroRNAs (miRNAs) are significant cancer biomarkers that can be used to assess an individual's BC risk [21]. They bind to target mRNAs' 3'‐untranslated region (3'UTR) in order to control gene expression. Through this binding, miRNAs regulate gene output by inducing the decay of target mRNAs or preventing their translation. Apoptosis, carcinogenesis, and numerous other biological and cellular processes, including cell division and proliferation, are all influenced by microRNAs (miRNAs) [22]. However, the function of miRNA is dependent on the location of miR‐SNPs. The SNPs located at the miRNA binding sites in regulatory regions of the gene significantly induce the gene function that contributes to the elevated BC risk [23].

In several genetic inheritance models, such as additive model 2 (OR = 2.48, CI = 1.12–5.48, *p* = 0.025), recessive (OR = 1.96, CI = 1.0–3.86, *p* = 0.051) and allelic (OR = 1.59, CI = 1.06–2.39, *p* = 0.026), the present study explored a strong connection of the miR‐218‐2 rs11134527 variant to the elevated BC risk. This finding strengthens the allelic and genotypic association of the variant with increased BC risk in Bangladeshi women. A previous study carried out in Iranian women also confirmed similar outcomes [24]. The connection between breast cancer susceptibility and this genetic variant was also examined in a prior meta‐analysis [22]. Jiao et al., however noted that the miRNA‐218‐2 (rs11134527) variant may not be considered a reliable breast cancer biomarker [25].

Furthermore, the susceptibility of various cancer types may be linked to the *miRNA‐218‐2* (rs11134527) variant. Previous research on populations from Bangladesh and China has connected this polymorphism to the risk of cervical cancer. Though this variant was linked to the elevated cervical cancer risk in the Bangladeshi female [26], it was connected to the decreased risk of cervical cancer in the Chinese women [27]. In addition, another investigation in the Chinese population failed to show any linkage of this SNP with oesophagal squamous cell carcinoma [28]. However, a meta‐analysis revealed a connection between the *miRNA‐218‐2* (rs11134527) polymorphism and a lower risk of cancer [29].

In line with the reduced MIR218‐2 expression observed in breast cancer tissues in our in‐silico analysis, previous functional studies provide mechanistic insight into how the rs11134527 variant may influence miR‐218 biogenesis. In addition to in‐silico predictions, experimental studies provide direct evidence that rs11134527 affects miR‐218 biogenesis. Genotype‐based expression analyses have demonstrated a distinct stepwise elevation in the mature miR‐218 levels among rs11134527 genotypes (AA < AG < GG), indicating a dose‐dependent regulatory influence on miRNA processing [30]. This effect is thought to result from increased structural stability of the pri‐miR‐218 hairpin, which facilitates more efficient Drosha/Dicer‐mediated cleavage. GTEx does not have genotype‐stratified miRNA expression data for rs11134527, but this is probably because large eQTL databases do not cover many miRNA‐SNP associations, not because they are not biologically important.

The clinicopathological features, diagnosis, and course of treatment of breast cancer patients in Bangladesh were also investigated in the current study. The most common clinicopathological characteristics of breast cancer patients, according to our research, are grade II breast cancer, invasive duct cell carcinoma, and positive ER, PR, and HER2 status. Aside from that, the most common diagnostic techniques used to find breast cancer are ultrasound and biopsy. Furthermore, the most widely used treatment for breast cancer is chemotherapy. The drugs that Bangladeshi breast cancer patients were prescribed the most were paclitaxel, doxorubicin, and cyclophosphamide. Previous studies involving breast cancer patients from Bangladesh also yielded similar results [2, 4, 20].

We faced some difficulties in carrying out this study that need to be discussed. Our study's sample size was not adequate to capture the true incidence of breast cancer in Bangladesh. Furthermore, the available information regarding diagnosis, treatment plan, and clinicopathological characteristics was insufficient to pinpoint the precise circumstances. Furthermore, the current study only included one SNP from this gene, which was inadequate to characterize the more detailed and extensive association between this gene and breast cancer risk. Thus, further research with a larger sample size and adequate clinicopathological data is needed to validate the results of our study.

## 5 Conclusions

With the exception of the over‐dominant model, the study demonstrated a strong association between the Bangladeshi population's elevated risk of breast cancer and the miRNA‐218‐2 (rs11134527) genetic variation. The results obtained from this investigation could potentially open up new avenues for genetic research. A larger sample size and adequate data on clinicopathological features, treatment course, and diagnosis patterns must be obtained for future research to validate the findings of the current study. Once confirmed, the research could aid in the advancement of personalized medicine.

## Author Contributions

**A. M. Shamim:** conceptualization, methodology, investigation, data curation, formal analysis, validation, writing –original draft. **Aiman Hossan:** methodology, investigation, data curation, validation, writing – original draft. **Md Shafiul Hossen:** software, formal analysis, data curation, validation, methodology, writing – review and editing, funding acquisition. **Md Abdul Barek:** methodology, investigation, data curation, writing – review and editing. **Mohammad A Rashid:** conceptualization, writing – review and editing, visualization, resources, project administration. **Mohammad Safiqul Islam:** conceptualization, supervision, methodology, project administration, writing – review and editing.

## Ethics Statement

The manuscript should state that informed consent was obtained from participants. Now they have mentioned that form was given to the participants.

## Conflicts of Interest

The authors declare no conflicts of interest.

## Transparency Statement

The lead author, Mohammad Safiqul Islam, affirms that this manuscript is an honest, accurate, and transparent account of the study being reported; that no important aspects of the study have been omitted; and that any discrepancies from the study as planned (and, if relevant, registered) have been explained.

## Supporting information


**Table S1:** Primer sequences used in the tetra‐primer ARMS‐PCR method. **Table S2:** PCR conditions of miR‐218‐2 rs11134527 polymorphism with respective fragments size.

## Data Availability

The data supporting this study's findings are available from the corresponding author upon reasonable request. The corresponding author takes complete responsibility for the integrity of the data and the accuracy of the data analysis.
