# Supplementary material for: Linkage Between miR‐218‐2 (rs11134527) Genetic Polymorphism and Breast Cancer Risk: A Case‐Control Study in the Bangladeshi Women
Source: Health Sci Rep. 2026 Apr 16;9(4):e72092. doi: 10.1002/hsr2.72092 (PMC13087437; doi:10.1002/hsr2.72092)
Supplement: Supplementary file 1 — Table S1: Primer sequences used in the tetra‐primer ARMS‐PCR method. Table S2: PCR conditions of miR‐218‐2 rs11134527 polymorphism with respective fragments size. [file HSR2-9-e72092-s001.docx]

**Supplementary tables**

**Table S1:** Primer sequences used in the tetra-primer ARMS-PCR method

| Primer | Sequence |
| --- | --- |
| Forward outer primer: | 5’- AGCGACTGGTCAGAGTCAAGGTCAGGAA-3’ |
| Reverse outer primer: | 5’-TTCTTTCCTTGGGAAGGAACCATGCTGT-3’ |
| Forward inner primer: | 5’-AAGCCGGAGCAGGCCCCCACTGATCA-3’ |
| Reverse inner primer: | 5’-TGGAACCCCACTCCTGATACTAATAAC-3’ |

**Table S2:** PCR conditions of miR-218-2 rs11134527 polymorphism with respective fragments size.

| Gene | PCR conditions | No. of cycles | Size of PCR product (bp) | Fragment sizes (bp) |
| --- | --- | --- | --- | --- |
| miR-218-2 rs11134527 | 95°C 1 min  57°C 30 s  72°C 30 s | 35 cycles | 280 | NH:GG: 180, 280  HE:GA: 180, 200, 280  MH: AA: 200, 280 |

Here, NH: Normal Homozygote; HE: Heterozygote; MH: Mutant Homozygote.
